# Cancer patterns in Iran: a gender-specific spatial modelling of cancer incidence during 2014–2017

**DOI:** 10.1186/s12885-024-11940-4

**Published:** 2024-02-12

**Authors:** Sharareh Faramarzi, Behzad Kiani, Shahla Faramarzi, Neda Firouraghi

**Affiliations:** 1 Department of Medical Informatics, School of Medicine, Mashhad University of Medical Sciences, Mashhad, Iran; 2 UQ Centre for Clinical Research, Faculty of Medicine, The University of Queensland, Brisbane, QLD Australia; 3 Department of Health Information Management School of Health Management and Information Sciences, Shiraz University of Medical Sciences, Shiraz, Iran

**Keywords:** Cancer incidence, Spatial epidemiology, Public health, SaTScan, Spatial scan statistics, Geographic Information Systems (GIS), Iran

## Abstract

**Background:**

Cancer is a significant public health concern and the second leading cause of death. This study aims to visualize spatial patterns of top common cancer types and identify high-risk and low-risk counties for these cancers in Iran from 2014 to 2017.

**Methods:**

In this study, we analyzed 482,229 newly diagnosed cancer cases recorded by the Iranian National Population-Based Cancer Registry from 2014 to 2017. We employed a purely spatial scanning model and local Moran *I* analysis to explore spatial patterns across Iran.

**Results:**

Approximately 53% of all cases were male. The average age of cancer diagnosis was 62.58 ± 17.42 years for males and 56.11 ± 17.33years for females. Stomach cancer was the most common cancer in men. The northern and northwestern regions of Iran were identified as high-risk areas for stomach cancer in both genders, with a relative risk (RR) ranging from 1.26 to 2.64 in males and 1.19 to 3.32 in females. These areas recognized as high-risk areas for trachea, bronchus, and lung (TBL) cancer specifically in males (RR:1.15–2.02). Central regions of Iran were identified as high-risk areas for non-melanoma skin cancers in both genders, ranking as the second most common cancer (RR:1.18–5.93 in males and 1.24–5.38 in females). Furthermore, bladder cancer in males (RR:1.32–2.77) and thyroid cancer in females (RR:1.88–3.10) showed concentration in the central part of Iran. Breast cancer, being the most common cancer among women (RR:1.23–5.54), exhibited concentration in the northern regions of the country. Also, northern regions of Iran were identified as high-risk clusters for colon cancer (RR:1.31–3.31 in males and 1.33–4.13 in females), and prostate cancer in males (RR:1.22–2.31). Brain, nervous system cancer, ranked sixth among women (RR:1.26–5.25) in central areas.

**Conclusions:**

The study's revelations on the spatial patterns of common cancer incidence in Iran provide crucial insights into the distribution and trends of these diseases. The identification of high-risk areas equips policymakers with valuable information to tailor targeted screening programs, facilitating early diagnosis and effective disease control strategies.

**Supplementary Information:**

The online version contains supplementary material available at 10.1186/s12885-024-11940-4.

## Background

Cancer is a complex and multifaceted disease that continues to be a critical global public health concern. Despite significant advances in cancer research and treatment, the lack of a universal preventative approach remains a significant challenge. The impact of cancer on individuals, families, and communities worldwide cannot be overstated, as it causes high levels of morbidity and mortality [1]. The burden of cancer is not limited to developed countries alone, but it also affects low- and middle-income countries, where resources for prevention, early detection, and treatment are often scarce. Approximately 70% of all cancer cases occur in low- and middle-income countries [2, 3]. It is predicted that the number of cancer cases in low- and middle-income countries will increase five-fold by 2030 [4]. In recent decades, developing countries have faced a growing challenge in providing equal access to healthcare services and managing the resources, Iran is no exception to this trend [5]. More than 50,000 new cases are diagnosed in the Iranian population each year [6]. Cancer poses a major health concern in Iran, as it is the second most prevalent chronic non-communicable disease and the third leading cause of death. There has been an increase in the number of new cases of cancer in recent years, highlighting the growing burden on the nation [7].

The environment has a direct impact on people's health, including the occurrence of cancer. Cancer patterns differ among populations and regions, primarily due to environmental factors such as industry environments [8], climate [9], socioeconomic status [10], access to healthcare [11], as well as physical activity [12], family history [13], age [14], and sex [15]. These factors collectively contribute to variations in cancer incidence rates and overall health outcomes. Studying cancer incidence patterns in specific areas can help identify clusters of high rates, revealing hypotheses about the link between environmental risk factors and localized cancer occurrences.

Early diagnosis of cancer can significantly reduce mortality rates. However, the high cost of screening procedures often makes it difficult for health systems, particularly in developing countries, to screen all individuals and detect cancer at an early stage [16]. Therefore, identifying high-risk areas is crucial for effective cancer screening and control policies. Spatial analysis, like the use of spatial scan statistics (SaTScan) and spatial autocorrelation, helps identify disease clusters and patterns. These methods are used to identify spatial clusters of disease incidence in geographic regions and offer valuable insights to guide policymakers in developing targeted interventions and improving public health [17, 18].

Many studies in Iran have conducted spatial analyses, typically focusing on the province scale or specific types of cancer. Kiani et al. have explored the geospatial patterns and analyzing data on cancer occurrence in Khorasan-Razavi Province [19]. Meanwhile, Babaee et al. examined geo-epidemiological of the most prevalent cancers at the provincial level in Iran in 2014 [20]. In a prior investigation, spatiotemporal patterns of gastric cancer incidence in Zanjan Province were examined. The findings indicate significant variations in the spatial distribution of gastric cancer incidence in the study area [21]. An additional investigation sought to examine the incidence patterns of high-risk clusters of breast and prostate cancers in Kerman Province between 2014 and 2017. The study observed a noticeably higher incidence rate in the northwestern region of Kerman, which could be used to develop customized screening and surveillance systems [22]. Another study utilized a spatial analysis to investigate the spatio-temporal distribution of colorectal cancer in the Iranian military community from 2007 to 2016 [23]. The study highlights the north and northwest regions of Iran as high-risk areas for this cancer, which should be considered in designing disease prevention [23].

To our knowledge, there is a lack of a comprehensive analysis of cancer incidence at the county level in the whole country. Cancer occurrence patterns vary widely across different geographic regions, and analyzing these patterns at the county level can offer valuable insights into the high-risk patterns. By identifying high-risk areas, public health officials can prioritize their efforts and allocate resources more effectively to prevent cancer and improve outcomes for affected individuals. To address this gap, we conducted a study to analyze the spatial patterns of top cancer incidence in Iran from 2014 to 2017 at the county level, with a focus on gender differences.

## Methods and materials

### Study area and population

**Fig. 1:**
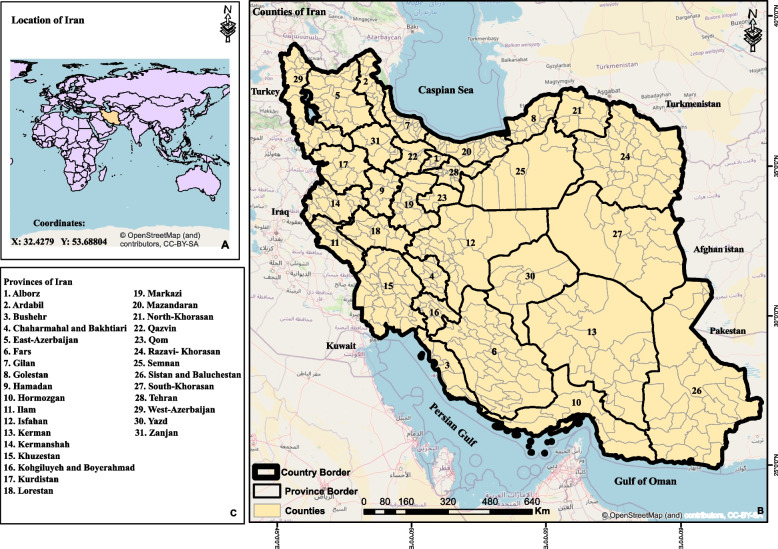
The map of the study area illustrates the geographical divisions regarding Iran, including the location of the country in the world, neighboring countries, and the borders of provinces and counties. The map also includes numbered labels indicating the names of provinces

This study was conducted in Iran, located in the western part of Asia. Iran shares western borders with Iraq and Turkey, and northern borders with Azerbaijan, Armenia, Turkmenistan, and the Caspian Sea. This country is also bounded to the east by Afghanistan and Pakistan, and to the south by the Persian Gulf and Oman Sea. Figure 1 presents a visual depiction of Iran's nationwide provinces. Iran consists of 423 counties and 31 provinces with a total area of 1,648,195 km and based on the last national census in 2016, the total population of Iran is approximately 80 million that including 40,333,417 men and 39,158,728 women [24].

### Data sources

In this study, we gathered the data from three different sources as follows:

1. Cancer cases data were obtained from the Iranian National Population-Based Cancer Registry (INPCR) [25]. The INPCR registers newly diagnosed cancer cases with malignant primary tumors, and regarding to metastatic cancers, only initial tumors are registered by this system. It uses national guideline and standard protocols of the International Agency for Research on Cancer (IARC) [26]. In this registry, the third edition of the International Classification of Diseases for Oncology (ICD-O) has been using for coding of tumor topography, morphology, behavior and grade [27]. The data includes patients’ characteristics such as age, gender, and patient’s address of residence; and tumor characteristics such as date of malignancy diagnosis, tumor topography and morphology. The INPCR started data gathering by using the data of the year 2014 and there are three to four years' delay in gathering and reporting data by the Iranian National Cancer Registry. Therefore, we analyzed the last reported incidence data for four years, between 2014 to 2017 at the county level, taking into account the residency county of the patients The main sources of data collection include pathology reports, clinical evaluation, cytology and death certificate. There were 482,229 cancer cases in the study area during studied period but we excluded 7 cases due to incomplete data.
2. Population data based on the last national census data in 2016 has been obtained from the statistical center of Iran [24].
3. The Ministry of the Interior supplied the shapefile format of county boundaries, represented as a vector map, reflecting the latest geopolitical division.

### Spatial scanning model

A retrospective spatial scanning analysis by Kulldorff was used to investigate statistically significant high-risk clusters [28] and determine whether cancer incidence was distributed randomly in the context of space. This analysis requires the information regarding the number of cases, population data, and the geographical coordinates of studied area [29]. A pre-defined circular scanning window, with a variable size, were developed across the space, to detect potential clusters without a prior assumption about their location or size. This window moved across the map, defines a set of zones and collect the number of observations inside the window to compare it with the number of cases outside. The relative risk (RR) was calculated by dividing the estimated risk within a cluster by the risk outside the cluster, assuming equal disease risk. The estimated risk was determined by dividing the number of observed cases by the expected cases. The expected number of events is determined based on the overall event rate in the study area [28].

In Kulldorff’s scan statistics, the first step is to determine a probability model and compute the likelihood ratio for each scanning window [28]. For each scanning window, the log likelihood ratio (LLR) was calculated, and it was used to rank the identified clusters. The cluster with the highest LLR was considered as most likely cluster and the others were known as secondary clusters. Overlapping clusters were not taken into account in this study. The significance of discovered clusters was determined using Monte Carlo simulation with 999 simulations to enhance reliability. The null hypothesis was rejected if the maximum LLR of the actual dataset fell within the top 5% of corresponding clusters in the random dataset, indicating significance at the 0.05 level.

In this study, we applied purely spatial cluster analysis in order to scan the space and discover spatial high-risk areas using discrete Poisson model. In this approach, the window size was set between 0 to 25% of population at risk. The generated spatial window scanned the entire studied area and identified potential clusters centered on coordinated points.

### Spatial autocorrelation

Spatial autocorrelation is an important concept in spatial analysis to understand the degree of similarity or dissimilarity between neighboring locations. In this study, the Local Moran *I* statistic was used to measure spatial autocorrelation. This statistic calculated a local indicator of spatial association for each county in the study area by comparing the cancer incidence at that location with the values of its neighboring features. This statistic values ranges from -1 to 1, with positive values indicating clustering groups of neighboring locations that exhibit similar characteristics, and negative values indicating outliers refer to locations that stand out due to their significantly different values compared to their neighboring locations. Outliers can reveal unique features where there is a high incidence rate that may not be classified as high-risk due to the difference with neighboring areas. However, these areas actually require more attention from policymakers to effectively controlling. This examination computes a z-score and *p*-value to determine if the observed patterns are more significant than what would be anticipated in a random arrangement [30]. In this method, the results are presented as follows:

- High-High (HH) clusters emphasize areas with high cancer incidence rate that are surrounded by other high-value areas. These areas are considered high-risk areas due to their concentration of high incidence rates.
- Low-Low (LL) clusters represent areas with low incidence rate that are surrounded by other low-value areas which show low-risk regions.
- High-Low (HL) outliers represent areas with high rate that are surrounded by low-value areas. These regions can raise alarm bells for policy makers to take notice and address the situation.
- Low–High (LH) outliers represent areas with low values that are surrounded with high-value areas. These areas are distinct because they have lower incidence rates despite being surrounded by regions with higher rates.

### Software

We used ArcGIS, v. 10.8 for data preparation, analyzing, and visualization [31].The SaTScan™ software, v 9.6.1 (Martin Kulldorff, Harvard Medical School, Boston and Information Management Services, Inc.) was used for spatial scan analysis [32].

## Results

### Descriptive statistical and spatial analysis

A total of 482,222 cancer cases were registered during 2014–2017, and 255,165 (53%) of these cases were males and 227,057 (47%) were females. The average age of cancer in men was 62.58 ± 17.42 and in women 56.11 ± 17.33. The most common cancers by incidence per 100,000 people, excluding non-melanoma skin cancer, in men were stomach (70.88), prostate (67.26), bladder (51.36), trachea, bronchus, lung (TBL) (43.87), and colon (41.27). In women, the top common cancers were breast (150.69), thyroid (40.18), stomach (35.27), colon (34.63), brain and nervous system (BNS) (20.57). Table 1 shows the number, percent, and incidence rate (per 100,000 people) of top 5 cancers and non-melanoma skin cancer for both genders. The descriptive map of most common cancers indicated that cancer incidence varied geographically across Iran. They can be found in the Supplementary File 1 (Figure S1 & S2).

**Table 1:** The number, percent, and incidence rate (per 100,000) of five most common cancers and non-melanoma skin cancer for both genders in Iran (2014 – 2017)

Gender	Cancer type	Number	Percent	Incidence (per 100,000 people)
**Male**	Stomach	28,592	11.20%	70.88
	Non-melanoma skin	28,025	10.98%	69.48
	Prostate	27,130	10.63%	67.26
	Bladder	20,719	8.1%	51.36
	Trachea, Bronchial, lung (TBL)	17,696	6.93%	43.87
	Colon	16,649	6.52%	41.27
**Female**	Breast	59,010	25.98%	150.69
	Non-melanoma skin	17,301	7.6%	44.18
	Thyroid	15,737	6.93%	40.18
	Stomach	13,814	6.08%	35.27
	Colon	13,562	5.97%	34.63
	Brain, Nervous, System (BNS)	8,056	3.54%	20.57

### Purely spatial and local Moral* I* analysis of most common cancers in males

**Fig. 2:**
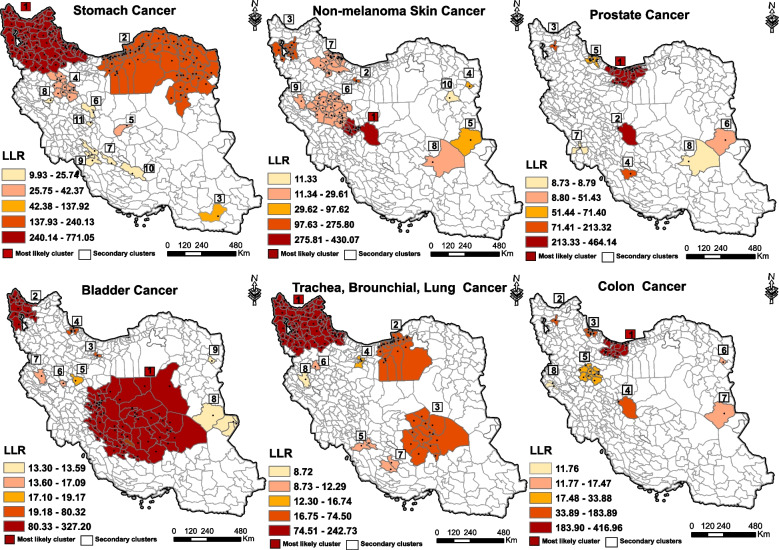
The figure showcases six maps illustrating the spatial patterns of six common cancers in males in Iran during the years 2014–2017. Each map is labeled with the name of the corresponding cancer. These maps have been created using a purely spatial scanning technique. The accompanying legend provides the classification of log likelihood ratio (LLR), which were employed to rank the identified clusters

**Fig. 3:**
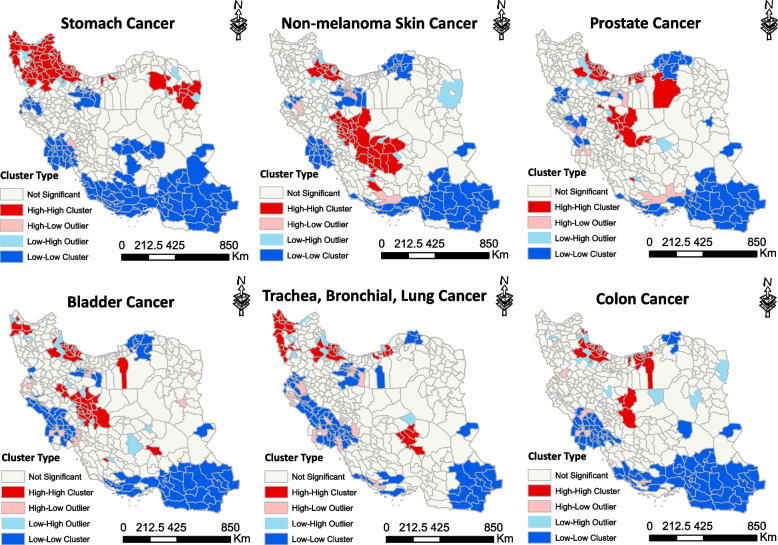
The figure represents six maps depicting the spatial distribution of six common cancers in males, created using Local Moran I analysis. Each map is labeled with the name of the corresponding cancer. The accompanying legend shows the cluster types for cancer incidence per 100,000 males in Iran during the period of 2014–2017. High-risk areas are depicted in red, low-risk areas in blue, areas with high-low incidence in pink, and areas with low–high incidence in light blue

Stomach cancer was the most common cancer among Iranian men with the highest incidence rates in the north and northwestern regions of Iran. Geographical scanning identified eleven significant high-risk clusters of stomach cancer. The RR for these clusters ranged from 1.26 to 2.64 (*P*-value < 0.05). The cluster with the highest LLR, considered as the most likely cluster (LLR: 771). Secondary high-risk clusters were located in northeastern and central regions (Fig. 2). Local Moran *I* analysis revealed HH clusters in the northwestern regions and some counties in the northeastern area. Low-risk areas were concentrated in the south, southwest, southeast and some counties of Kermanshah province (Fig. 3).

Non-melanoma skin cancer ranked second among the most prevalent cancers in both Iranian men and women. Spatial analysis identified ten significant high-risk clusters of non-melanoma skin cancer in men. The RR for these clusters ranged from 1.18 to 5.93. The cluster with the highest LLR (LLR: 430.07) was located in Isfahan, Chaharmahal and Bakhtiari (Fig. 2). According to the findings of the local Moran's analysis, central regions were identified as high-risk areas and low-risk areas were focused on the southeast parts of the country (Fig. 3).

Prostate cancer was a prevalent form of cancer among men, ranking third in terms of incidence. In Iran, spatial analysis has highlighted eight significant high-risk clusters of prostate cancer. Based on spatial analysis, Tehran and Mazandaran provinces have been identified as the most high-risk clusters for prostate cancer (Fig. 2). The RR for these clusters ranged from 1.22 to 2.31 (*P*-value < 0.05). Other clusters were scattered in different regions of Iran. Figure 3 shows that based on Moran's local analysis, the majority of counties in the north and central regions were identified as high-high clusters, indicating a significant concentration of high cancer incidence. However, there were some outliers in the south and western areas (HL), which had high incidence rates but were not classified as high-risk areas. These outliers can serve as an alarm for policymakers, signaling the need for proactive measures and policies.

Bladder cancer was the fourth most common cancer for men, and it has a particularly high incidence rate in the central and northwestern regions of Iran. Geographical scanning identified nine significant high-risk clusters of bladder cancer. The RR for these clusters ranged from 1.32 to 2.77 (*P*-value < 0.05). The most likely cluster (LLR: 327.20) was located in the provinces of Fars, Kerman, Yazd and Isfahan. Secondary high-risk clusters were scattered in the north, northwestern, and west of Iran (Fig. 2). Moran analysis revealed that both the northwest and central areas of Iran involved by high-risk clusters. The regions with the highest concentration of LL clusters were primarily located in the west, and southeast (Fig. 3).

Trachea, bronchial, lung cancer was ranked as the fifth most common cancer among men. Spatial analysis indicated eight significant high-risk clusters. The northwest areas of Iran were identified as hotspot areas for TBL. The RR for these clusters ranged from 1.15 to 2.02 (*P*-value < 0.05). The most likely cluster (LLR: 242.72) was located in the provinces of West Azerbaijan and East Azerbaijan, Zanjan, Ardabil and Gilan. Most of the secondary clusters were located in Golestan, Mazandaran, Semnan, Kerman and Yazd provinces (Fig. 2). Moran *I* revealed that the northwestern and northern areas, along with some counties within Kerman province, were focal points of HH clusters. Low-Low clusters were focused on the southeast and west of Iran (Fig. 3).

Among men, colon cancer was the sixth most frequently diagnosed cancer, with the highest incidence rates observed in the north of Iran. Spatial analysis identified a total of eight significant high-risk clusters, with relative risks ranging from 1.31 to 3.31 and a statistically significant *p*-value (< 0.05). The cluster with the highest LLR value of 416.95 was identified in Mazandaran, Alborz and Tehran provinces (Fig. 2). Secondary clusters are scattered in different areas. Based on Moran's local analysis, a number of northern counties and some parts of Isfahan were identified as hotspots clusters. The highest concentration of cold spots was observed in the south, southeast and western regions (Fig. 3). Each number within Fig. 2 represents the order of clusters with *p*-value < 0.05. Detailed information about each cluster can be found in Supplementary File 2 (Table S1).

### Purely spatial and local Moran *I* analysis of most common cancers in females

**Fig. 4:**
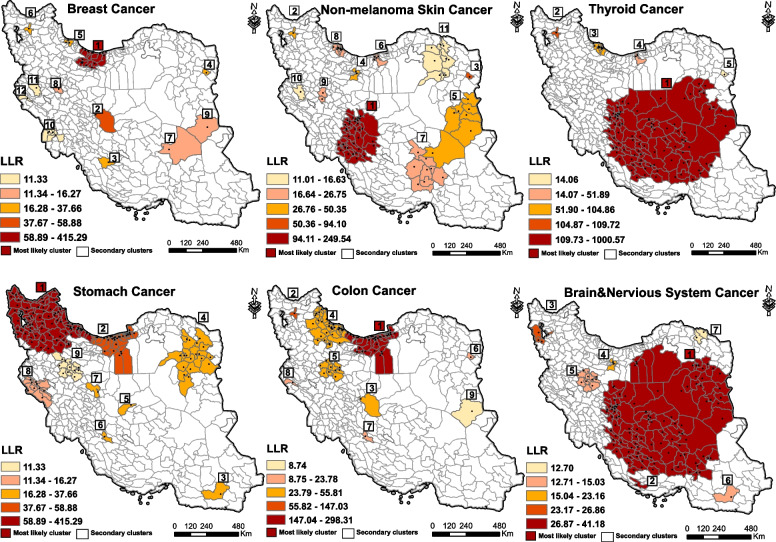
The figure showcases six maps illustrating the spatial patterns of six common cancers in females in Iran during the years 2014–2017. Each map is labeled with the name of the corresponding cancer. These maps have been created using a purely spatial scanning technique. The accompanying legend provides the classification of log likelihood ratio (LLR), which were employed to rank the identified clusters

**Fig. 5:**
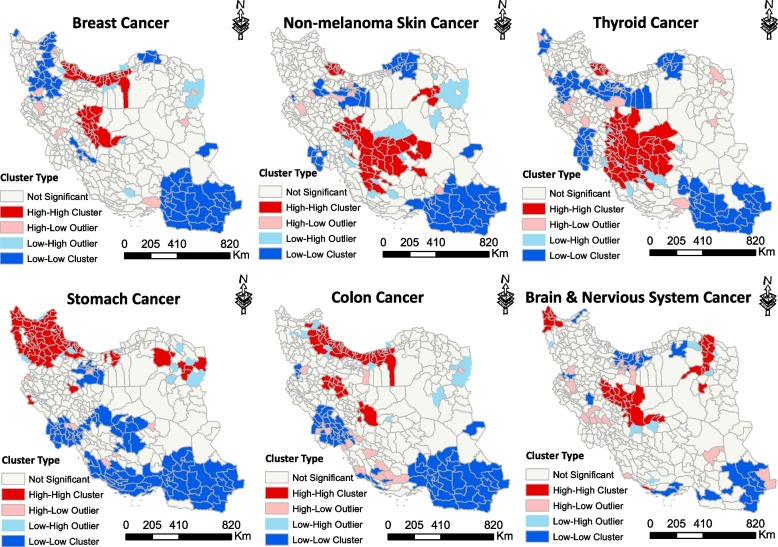
The figure represents six maps depicting the spatial distribution of six common cancers in females, created using Local Moran I analysis. Each map is labeled with the name of the corresponding cancer. The accompanying legend shows the cluster types for cancer incidence per 100,000 females in Iran during the period of 2014–2017. High-risk areas are depicted in red, low-risk areas in blue, areas with high-low incidence in pink, and areas with low–high incidence in light blue

Breast cancer was the most common cancer among women in Iran. Through spatial analysis, we have identified twelve significant high-risk clusters of breast cancer. The RR for these clusters ranged from 1.23 to 5.54, with a *P*-value less than 0.05. Among these clusters, the most likely cluster, with an LLR of 1228.36, predominantly encompassed the northern regions of the country (Fig. 4). Secondary clusters were observed in different parts of Iran. Furthermore, applying Moran's local analysis revealed that the high-risk areas encompassed the northern and central regions and northwestern areas, along with southeastern part were identified as LL clusters (Fig. 5).

The central regions of Iran have been identified as high-risk areas for non-melanoma skin cancer. Among women, the highest incidence rate (LLR: 249.53) was observed in these regions through purely spatial analysis. There were more secondary clusters in the northeast, east, and southeast regions, with RR ranges from 1.24 to 5.38 (Fig. 4). Local Moran's examination revealed that the high-risk areas have been identified in the central regions (Fig. 5).

Among women, thyroid cancer was the third most common type of cancer with the highest incidence occurring in the central, south central and east central regions of Iran. Spatial analysis indicated the presence of five significant high-risk clusters, with relative risks ranging from 1.88 to 3.10 (*P*-value < 0.05). The most likely cluster with LLR of 1000.57 was located in Yazd, Isfahan, Fars and Kohgiluyeh and Boyer Ahmad provinces. There were secondary clusters in the provinces of Gilan, Mazandaran, Tabriz and Bakherz counties (Fig. 4). Moran *I* modelling revealed HH clusters in the central part and LL clusters in southeastern and some western counties (Fig. 5). Stomach cancer was a prevalent cancer among Iranian women, ranking as the fourth most common type. In this study we found nine significant high-risk clusters with RR ranges 1.19 to 3.32. The northwestern regions and parts of the northern counties were identified as most likely cluster (LLR: 415.28). Most of the secondary clusters were located in the northern, western and northeastern of Iran (Fig. 4). Local Moran *I* results regarding high-risk areas were consistent with purely spatial analysis and this model identified most of the southern and southwestern areas as low-risk clusters (Fig. 5).

Colon cancer ranked as the fifth most prevalent cancer among women. Spatial analysis revealed that the northern regions of Iran exhibit the highest risk cluster, displaying the highest incidence of colon cancer between 2016–2017. The most likely cluster, with a LLR of 298.31, was located Tehran, Golestan, Mazandaran and, Semnan provinces (Fig. 4). Similarly, in terms of local Moran analysis, high-risk areas have been concentrated in the northern counties and low-risk areas in the southeastern and western regions. Southwestern counties were involved by high-low outliers (Fig. 5).

Brain and nervous system cancer was the sixth most common cancer among Iranian women. Purely spatial identified high-risk areas in the south central, central and eastern areas. Spatial analysis indicated seven significant high-risk clusters. The RR for these clusters ranged from 1.26 to 5.25. Some of the secondary clusters were located in Hamadan, and West Azerbaijan provinces (Fig. 4). Moran's analysis showed that there were HH clusters in the central, eastern, and northwestern areas, and it also identified high-low outliers in various parts of Iran (Fig. 5). Each number in the box in Fig. 4 represents the order of clusters with a *p*-value < 0.05. Additional information about each cluster can be found in Supplementary File 2 (Table S2).

## Discussion

We have investigated the spatial patterns of six common cancer incidence among Iranian men and women for the years 2014–2017, utilizing data from the Iranian population-based cancer registry. This study represents the first attempt to explore the spatial high and low-risk clusters of common cancers in Iran at the county level, while also accounting for gender differences. Based on the results, the number of cancer cases in men was higher than in women and the average age of cancer in women is lower than in men. The findings revealed that various types of cancer exhibited regional variations, with specific regions in Iran being identified as high-risk areas for common cancer types.

Comparing the incidence and mortality rates of cancers in 2020 in Iran reveals that the rankings differ. The position in the mortality rank does not completely overlap with that in the incidence rank. This is because the death rate is noticeably higher for certain types of malignancies compared to others [33]. Stomach, lung, and liver (including trachea and bronchus) remain the top three deadliest cancers in the general population. On the other hand, prostate and thyroid cancers have the most favorable prognosis, with a 5-year survival rate close to 100% [34]. The incidence of cancer is influenced by the rates of detection and diagnosis. If a region has efficient screening programs and accessible healthcare, they may identify and report more cancer cases, resulting in a higher incidence rate. On the other hand, lower mortality rates may be attributed to early detection and effective treatment options. Early detection of breast cancer plays a crucial role in improving patients' prognosis and reducing mortality among women [35].

A recent study by Roshandel et al., revealed that the incidence of cancer in Iran is on the rise and currently exceeds the global average [36]. Different factors can contribute to an increasing trend in cancer incidence. Stomach cancer was the most common cancer among men and the fourth most common cancer among women. Previous studies have indicated that the northwestern regions of Iran exhibit a higher prevalence of stomach cancer among both men and women. This observation was likely attributed to the presence of common risk factors shared by individuals in these areas. One such risk factor is Helicobacter pylori infection [37]. Numerous studies have demonstrated a significant prevalence of this infection in these areas [38, 39]. In Ardabil Province, the prevalence of Helicobacter pylori infection was found to be over 89% among individuals aged 40 years old and above [40]. The usage of biomass for cooking and heating in the cold climate of northwestern Iran is another contributing factor to the high incidence of stomach cancer in this region. This is due to the increased exposure of residents to polycyclic aromatic hydrocarbons, which have been identified as a risk factor for stomach cancer, according to studies [40]. Tobacco use, including cigarettes smoking, as well as a high intake of salt and red meat, low consumption of antioxidants, poor oral and dental hygiene, and opium use are all considered as risk factors for stomach cancer [41–44]. Based on our study we have found that men are more likely to develop stomach cancer than women. One possible explanation for this is that women tend to have higher levels of estrogen hormone compared to men, and this hormone has been shown to have a protective effect against the development of stomach cancer in women [45].

Based on our results, non-melanoma skin cancer was the second common cancer among both men and women. Studies indicated that this type of cancer was more common in men than in women and was more commonly observed in older age groups and increasing incidence rates reported in Iran [46, 47]. Skin cancer is primarily caused by exposure to ultraviolet radiation [48]. People who are frequently exposed to sunlight have a higher risk of skin cancer than those who are less exposed to light [49]. Men, due to their occupations, tend to be more exposed to sunlight, resulting in a higher incidence of skin cancer among men compared to women. In our study, Isfahan and Chaharmahal and Bakhtiari Provinces had the highest incidence rates of skin cancer. Sun exposure is a major risk factor for skin cancer worldwide, and Isfahan province has a sunny and dry climate with high UV radiation levels. Lumpy skin disease (LSD) is a transmissible disease in cattle that causes skin lesions on the human body. This disease is transmitted through a virus that causes skin cancer in humans. According to a study conducted by Gehsareh Ardestani and colleagues, this disease was prevalent in Chaharmhal and Bakhtiari province, and the results of this study showed that there is a risk of contracting LSD in this province [50]. Natural background radiation is the radiation that comes from natural sources, such as cosmic rays, rocks, soil, water, and air. A study by Mortazavi et al. found that Chaharmahal and Bakhtiari province had one of the highest levels of natural background radiation in Iran, and suggested that this could increase the risk of skin cancer in the local population [51].

Prostate cancer was a prevalent cancer in men, ranking as the third most common. Smoking has been identified as the most significant cause of prostate cancer [52]. The incidence rate of prostate cancer varies geographically and this indicates the impact of environmental factors on its occurrence. For instance, arsenic compounds classified by WHO as cancer-causing agents in prostate cancer cases. There has been an increase in prostate cancer cases in Iran [53]. Therefore, promoting a healthy lifestyle and regular screening programs could potentially reduce the incidence of prostate cancer.

Bladder cancer was more commonly diagnosed in men, with its incidence being four times higher in men than women [19]. Recent studies suggest an upward trend in bladder cancer incidence rates [54]. The two primary risk factors for bladder cancer are smoking and age [54–56], where smokers are at a four-fold increased risk of developing bladder cancer [57, 58]. Smoking is particularly prevalent in the provinces of Yazd and West Azerbaijan which also have a high incidence of bladder cancer, indicating that smoking may be linked to the higher occurrence of this disease in these region [59, 60]. Opium consumption has also been shown to increase the risk of bladder cancer, and Kerman Province is one of the areas with high opium usage and subsequent higher incidence of bladder cancer [61].

Lung cancer was a significant health concern in Iran, with it being the fifth most common cancer among men and the second most deadly cancer in many parts of the country [62]. Research shows that men are more likely to be diagnosed with lung cancer than women, which can be attributed to the higher rates of smoking among men [63, 64]. Other risk factors for lung cancer include air pollution, hookah use and opium consumption [64]. According to our study, northwestern regions of Iran have a higher incidence of lung cancer. Previous studies have shown that the West Azerbaijan province has been identified as having high rates of smoking [59].

Among women, Breast cancer was the first most common type of cancer. It has been observed that Iranian women tend to be diagnosed with this disease approximately ten years earlier than women in developed nations [65]. According to our study, Tehran, Alborz, and Mazandaran provinces have the highest incidence of breast cancer. Additionally, the provinces of Isfahan, Fars, Yazd, and Kerman also show a high incidence rate following these regions. Air pollution and higher exposure to carcinogens in urban areas might be contributing to the increased number of diagnosed breast cancer cases in these provinces. [66–68]. Factors such as smoking, lack of physical activity, an unhealthy lifestyle, and having a body mass index over 30 are known to be associated with an increased risk of developing breast cancer [69, 70]. With the decline in fertility rates among women, it is predicted that the number of breast cancer cases will double by 2030 [71]. Isfahan has a lower marriage rate compared to other provinces, which may contribute to the high incidence of breast cancer in the region. Moreover, many women in Isfahan are unaware of breast cancer symptoms. [72]. The aforementioned findings underscore the importance of implementing awareness programs in high-risk areas to bridge the information gap and, consequently, reduce the burden of breast cancer [35].

Thyroid cancer was currently the third most common type of cancer among women. Recent studies have shown a significant rise in the incidence of thyroid cancer [36]. Based on our results, the provinces of Fars, Kerman, Yazd, Isfahan, Kohgiluyeh and Boyer Ahmad, Chaharmahal and Bakhtiari were identified as high-risk areas. Obesity is one of the known risk factors for thyroid cancer. According to a study conducted by Djalalinia et al., they examined the pattern of obesity and overweight in the country and among two groups of women and men, and in the women's group, Yazd, Kerman, Kohgiluyeh and Boyer Ahmad, Chaharmahal and Bakhtiari provinces were identified as high-risk areas [73]. Studies have shown that provinces with a higher prevalence of obesity also tend to have a higher incidence of thyroid cancer [74, 75]. A history of exposure to radiation in the head and neck area can increase the risk of thyroid cancer[76]. According to a study conducted by Dr. Shahbazi in the Chaharmahal and Bakhtiari provinces, it has been found that the presence of background radiation in this region is associated with an elevated risk of developing thyroid cancer [51].

According to our findings, the high-risk areas for BNS cancer were in the provinces of Kerman, Fars, Semnan, South Khorasan, Razavi Khorasan, Kohgiluyeh and Boyer-Ahmad, Isfahan, Chahamhal and Bakhtiari and Yazd. The cause of central nervous system malignancies is generally unknown [77]. Over the past few decades, extensive epidemiologic research has been dedicated to identifying environmental factors that contribute to the development of brain tumors. Regrettably, despite these efforts, significant advancements in this area have been limited. According to a study by Oloumi.et al. lead, cadmium, arsenic, thallium mercury increase the risk of BNS cancer [78]. Salari et al. conducted a study that provides evidence supporting the presence of lead in the soil of Kerman province. This finding can be attributed to several factors, including the existence of lead mines and industrial areas within the province [79]. Furthermore, another study focused on assessing the concentration of toxic heavy metals in the blood of residents in Kerman. This investigation aimed to determine the potential exposure levels and impacts of such metals on the local population [80]. Additionally, a study conducted in Isfahan province revealed the presence of lead and cadmium in both groundwater and soil samples within the region. This investigation shed light on the environmental contamination of these heavy metals in Isfahan province [80]. In Yazd province, the amount of lead is higher than normal [81, 82]. Other potential risk factors have been identified, including exposure to electromagnetic fields, environmental factors, occupational hazards, and long-term exposure to cigarette smoke, particularly among the spouses and children of smokers [83]. According to a study conducted by Sohrabi et al. in 2011 in the provinces of Fars, Chaharmahal, Bakhtiari, Kohgiluyeh, and Boyer Ahmad, smoking was the highest among men [84].

Comprehensive information about cancer incidence enables policymakers to make informed decisions regarding screening and treatment enhancements. Knowing where high-risk areas are located allows tailored interventions for specific populations. By prioritizing prevention, specialized care, and supportive environments, policymakers aim to enhance rehabilitation services in early detection, treatment outcomes, and the quality of life for cancer-affected individuals. Ultimately, the goal is to achieve evidence-based comprehensive information for effective policymaking.

## Limitation

The cancer registration office in Iran had outdated information that was reported with a delay of 3 to 4 years. The accuracy of data recording was also compromised due to the absence of accurate electronic recording, leading to errors during data entry. Another limitation was the lack of an annual census in Iran; instead, we used the results of the latest census conducted in 2016. As well, there are some limitations about Kulldroff scan statistics. This method assumes that the disease cluster shapes follow predefined geometric forms, such as circles or ellipses. However, real disease clusters can have irregular shapes, making it more difficult for the method to accurately detect them. Also, it relies on a defined maximum cluster size parameter that determines the maximum number of cases that can be considered within a cluster. Selecting an appropriate cluster size can be subjective, and different choices may lead to varying results. It's important to note that while the Kulldorff SaTScan method has limitations, it is still a widely-used and valuable tool for disease cluster detection and spatial epidemiology analysis.

## Conclusion

The study's findings on the geospatial patterns of common cancer incidence in Iran offer valuable insights into the distribution of these diseases. Stomach, non-melanoma skin, and colon cancers were common among both women and men, and the spatial patterns in both genders were similar. By identifying high-risk areas, policymakers can more effectively design targeted screening programs that facilitate early diagnosis and disease control. Such initiatives can help reduce the burden of cancer by facilitating timely interventions and improving healthcare outcomes. This study provides a fundamental assessment that can help generate hypotheses regarding associations between risk factors and high-risk areas for future studies.

### Supplementary Information

**Additional file 1:** **Figure S1.** The incidence rate maps of common cancers in males in Iran (2014 – 2017). **Figure S2.** The incidence rate maps of common cancers in women in Iran (2014 – 2017).

** Additional file 2:** **Table S1.** Information of high-risk purely spatial clusters of common cancers in males, in Iran during 2014-2017. **Table S2.** Information of high-risk purely spatial clusters of common cancers in females, in Iran during 2014-2017.

** Additional file 3.**

## Data Availability

The data analysed in the study are available as Supplementary File 3.

## Abbreviations

TBL: Trachea, Bronchus, Lung
BNS: Brain, Nervous System
GIS: Geographic Information Systems
INPCR: Iranian National Population-Based Cancer Registry
IARC: International Agency for Research on Cancer
ICD-O: International Classification of Diseases for Oncology
RR: Relative Risk
LLR: Log Likelihood Ratio
LSD: Lumpy skin disease
