# Supplementary material for: Cancer patterns in Iran: a gender-specific spatial modelling of cancer incidence during 2014–2017
Source: BMC Cancer. 2024 Feb 12;24:191. doi: 10.1186/s12885-024-11940-4 (PMC10860283; doi:10.1186/s12885-024-11940-4)
Supplement: Supplementary file 1 — Additional file 1: Figure S1. The incidence rate maps of common cancers in males in Iran (2014 – 2017). Figure S2. The incidence rate maps of common cancers in women in Iran (2014 – 2017). [file 12885_2024_11940_MOESM1_ESM.docx]

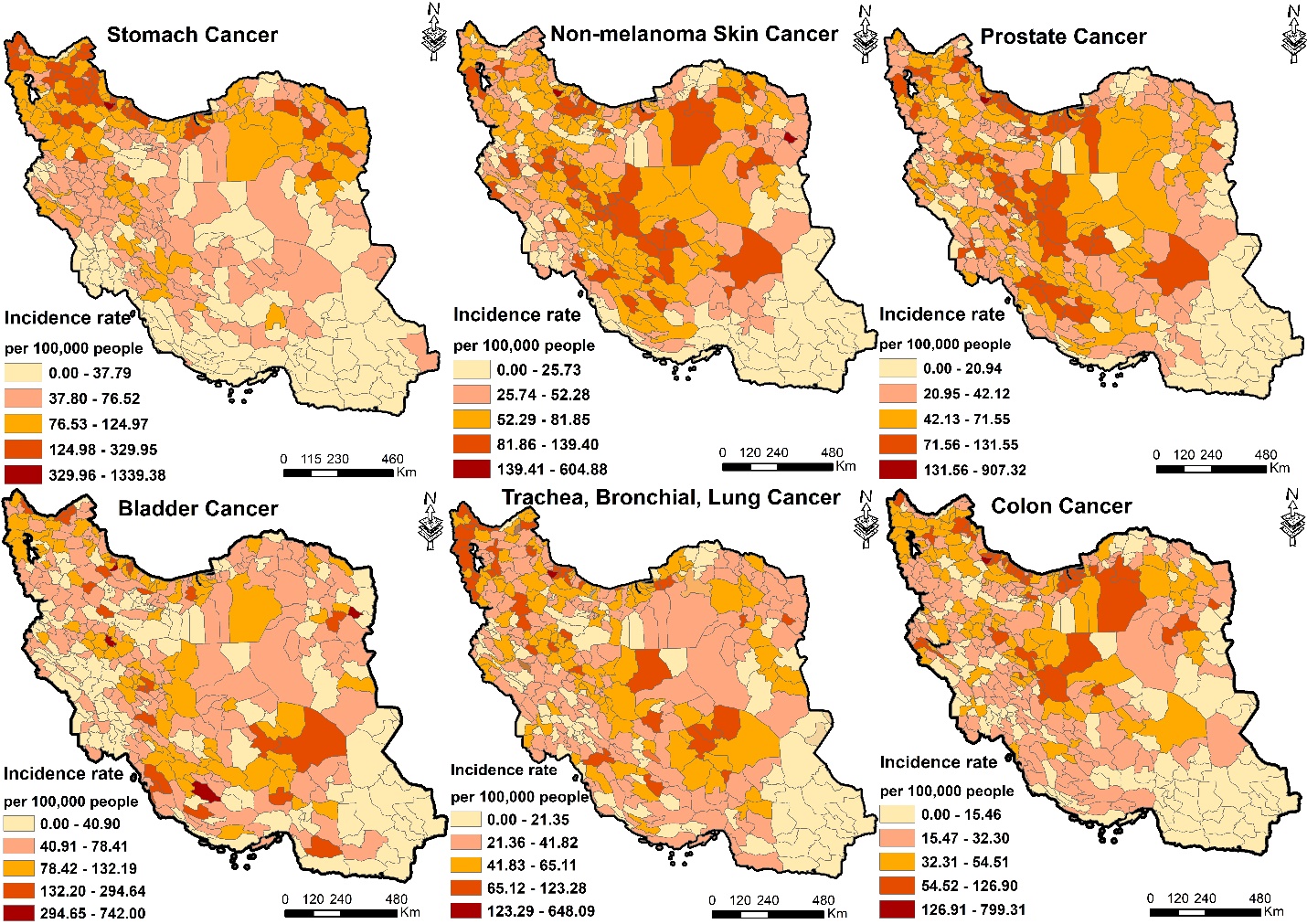


FigureS1: The incidence rate maps of common cancers in males in Iran (2014 – 2017)


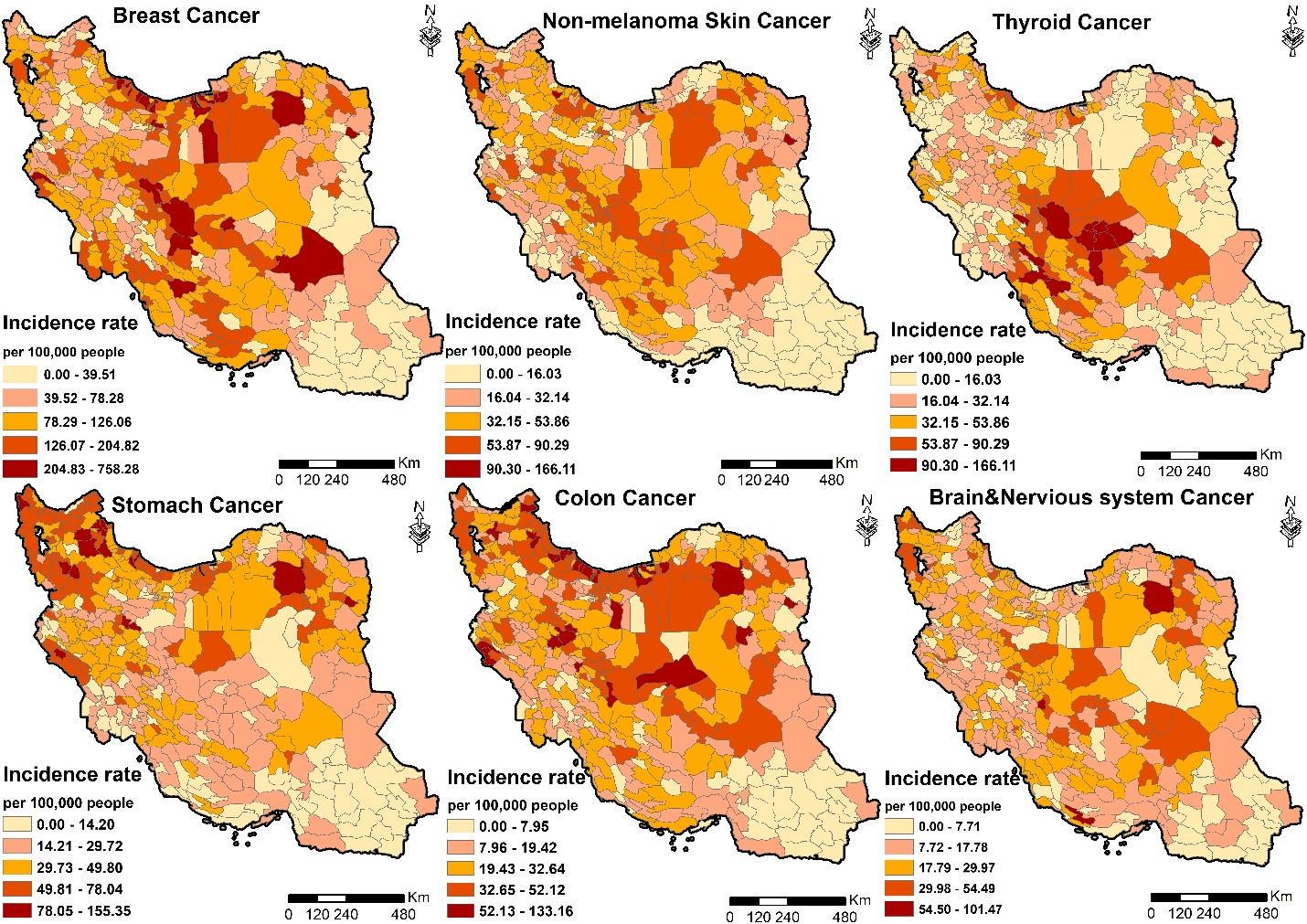


FigureS2: The incidence rate maps of common cancers in women in Iran (2014 – 2017)
