# Supplementary material for: Cancer patterns in Iran: a gender-specific spatial modelling of cancer incidence during 2014–2017
Source: BMC Cancer. 2024 Feb 12;24:191. doi: 10.1186/s12885-024-11940-4 (PMC10860283; doi:10.1186/s12885-024-11940-4)
Supplement: Supplementary file 2 — Additional file 2: Table S1. Information of high-risk purely spatial clusters of common cancers in males, in Iran during 2014-2017. Table S2. Information of high-risk purely spatial clusters of common cancers in females, in Iran during 2014-2017. [file 12885_2024_11940_MOESM2_ESM.docx]

Table S1: Information of high-risk purely spatial clusters of common cancers in males, in Iran during 2014-2017

| **Stomach** | **Cluster** | **Population** | **Number of cases** | **Expected cases** | **Observed / expected** | **RR** | **LLR** | **P-value** |
| --- | --- | --- | --- | --- | --- | --- | --- | --- |
|  | **#1** | 6629460 | 6991 | 4442.13 | 1.57 | 1.77 | 771.04 | 0.00 |
|  | **#2** | 4459368 | 4177 | 2988.04 | 1.40 | 1.47 | 240.13 | 0.00 |
|  | **#3** | 227133 | 398 | 152.19 | 2.62 | 2.64 | 137.91 | 0.00 |
|  | **#4** | 2245705 | 1864 | 1504.76 | 1.24 | 1.26 | 42.36 | 0.00 |
|  | **#5** | 94355 | 135 | 63.22 | 2.14 | 2.14 | 30.72 | 0.00 |
|  | **#6** | 46461 | 79 | 31.13 | 2.54 | 2.54 | 25.74 | 0.00 |
|  | **#7** | 124646 | 149 | 149 | 1.78 | 1.79 | 20.84 | 0.00 |
|  | **#8** | 113925 | 138 | 76.34 | 1.81 | 1.81 | 20.11 | 0.00 |
|  | **#9** | 245231 | 234 | 164.32 | 1.42 | 1.43 | 13.13 | 0.00 |
|  | **#10** | 70275 | 81 | 47.09 | 1.72 | 1.72 | 10.04 | 0.01 |
|  | **#11** | 84195 | 93 | 56.42 | 1.65 | 1.65 | 9.92 | 0.01 |
| **Non-melanoma skin** | **#1** | 1901347 | 2313 | 1200.71 | 1.93 | 2.02 | 430.07 | 0.00 |
|  | **#2** | 4347534 | 3976 | 2745.50 | 1.45 | 1.53 | 275.79 | 0.00 |
|  | **#3** | 1953981 | 1946 | 1233.95 | 1.58 | 1.62 | 185.02 | 0.00 |
|  | **#4** | 27580 | 103 | 17.42 | 5.91 | 5.93 | 97.62 | 0.00 |
|  | **#5** | 334843 | 398 | 211.46 | 1.88 | 1.90 | 65.85 | 0.00 |
|  | **#6** | 3321130 | 2443 | 2097.31 | 1.16 | 1.18 | 29.60 | 0.00 |
|  | **#7** | 1901531 | 1468 | 1200.83 | 1.22 | 1.24 | 29.20 | 0.00 |
|  | **#8** | 375918 | 354 | 237.39 | 1.49 | 1.50 | 25.11 | 0.00 |
|  | **#9** | 548326 | 476 | 346.27 | 1.37 | 1.38 | 22.06 | 0.00 |
|  | **#10** | 44647 | 57 | 28.19 | 2.02 | 2.02 | 11.33 | 0.00 |
| **Prostate** | **#1** | 6286591 | 5521 | 3716.28 | 1.49 | 1.63 | 464.13 | 0.00 |
|  | **#2** | 1133446 | 1491 | 670.03 | 2.23 | 2.31 | 386.36 | 0.00 |
|  | **#3** | 897157 | 1064 | 530.35 | 2.01 | 2.05 | 213.32 | 0.00 |
|  | **#4** | 942444 | 1072 | 557.12 | 1.92 | 1.97 | 192.48 | 0.00 |
|  | **#5** | 771941 | 731 | 456.33 | 1.60 | 1.62 | 71.39 | 0.00 |
|  | **#6** | 334843 | 356 | 197.94 | 1.80 | 1.81 | 51.42 | 0.00 |
|  | **#7** | 656830 | 473 | 388.28 | 1.22 | 1.22 | 8.78 | 0.03 |
|  | **#8** | 375918 | 287 | 222.22 | 1.29 | 1.30 | 8.72 | 0.03 |
| **Bladder** | **#1** | 5649604 | 3869 | 2589.08 | 1.49 | 1.63 | 327.20 | 0.00 |
|  | **#2** | 2089854 | 1726 | 957.73 | 1.80 | 1.88 | 265.42 | 0.00 |
|  | **#3** | 4347534 | 2547 | 1992.37 | 1.28 | 1.32 | 80.32 | 0.00 |
|  | **#4** | 614852 | 515 | 281.77 | 1.83 | 1.85 | 78.85 | 0.00 |
|  | **#5** | 300245 | 216 | 137.60 | 1.57 | 1.58 | 19.17 | 0.00 |
|  | **#6** | 163828 | 131 | 75.08 | 1.74 | 1.75 | 17.08 | 0.00 |
|  | **#7** | 548326 | 344 | 251.29 | 1.37 | 1.38 | 15.55 | 0.00 |
|  | **#8** | 455602 | 288 | 208.79 | 1.38 | 1.39 | 13.59 | 0.00 |
|  | **#9** | 27580 | 35 | 12.64 | 2.77 | 2.77 | 13.30 | 0.00 |
| **TBL** | **#1** | 5571639 | 3330 | 2295.31 | 1.45 | 1.56 | 242.73 | 0.00 |
|  | **#2** | 1793722 | 1085 | 738.95 | 1.47 | 1.50 | 74.50 | 0.00 |
|  | **#3** | 876851 | 595 | 361.23 | 1.65 | 1.67 | 64.85 | 0.00 |
|  | **#4** | 4534757 | 2108 | 1868.15 | 1.13 | 1.15 | 16.73 | 0.00 |
|  | **#5** | 295258 | 180 | 121.64 | 1.48 | 1.49 | 12.28 | 0.00 |
|  | **#6** | 70865 | 59 | 29.19 | 2.02 | 2.02 | 11.73 | 0.00 |
|  | **#7** | 223336 | 141 | 92.01 | 1.53 | 1.54 | 11.27 | 0.00 |
|  | **#8** | 548326 | 291 | 225.89 | 1.29 | 1.29 | 8.72 | 0.02 |
| **Colon** | **#1** | 6205400 | 3764 | 2380.39 | 1.58 | 1.77 | 416.95 | 0.00 |
|  | **#2** | 897157 | 750 | 344.15 | 2.18 | 2.24 | 183.89 | 0.00 |
|  | **#3** | 676082 | 493 | 259.34 | 1.90 | 1.93 | 84.82 | 0.00 |
|  | **#4** | 1133446 | 715 | 434.79 | 1.64 | 1.68 | 78.07 | 0.00 |
|  | **#5** | 2125655 | 1054 | 815.40 | 1.29 | 1.31 | 33.88 | 0.00 |
|  | **#6** | 27580 | 35 | 10.58 | 3.31 | 3.31 | 17.47 | 0.00 |
|  | **#7** | 334843 | 200 | 128.45 | 1.56 | 1.56 | 17.17 | 0.00 |
|  | **#8** | 144160 | 95 | 55.30 | 1.72 | 1.72 | 11.75 | 0.00 |

Table S2: Information of high-risk purely spatial clusters of common cancers in females, in Iran during 2014-2017

| **Breast** | **Cluster** | **Population** | **Number of cases** | **Expected cases** | **Observed / expected** | **RR** | **LLR** | **P-value** |
| --- | --- | --- | --- | --- | --- | --- | --- | --- |
|  | **#1** | 6193679 | 12953 | 8502.98 | 1.52 | 1.69 | 1228.36 | 0.00 |
|  | **#2** | 1109803 | 2975 | 1523.59 | 1.95 | 2.01 | 559.73 | 0.00 |
|  | **#3** | 926557 | 2015 | 1272.02 | 1.58 | 1.61 | 189.22 | 0.00 |
|  | **#4** | 27035 | 205 | 37.11 | 5.52 | 5.54 | 182.72 | 0.00 |
|  | **#5** | 610838 | 1386 | 1386 | 1.65 | 1.67 | 151.83 | 0.00 |
|  | **#6** | 875876 | 1794 | 1202.45 | 1.49 | 1.51 | 129.54 | 0.00 |
|  | **#7** | 362806 | 835 | 498.08 | 1.68 | 1.69 | 95.56 | 0.00 |
|  | **#8** | 1679058 | 2957 | 2305.09 | 1.28 | 1.30 | 88.69 | 0.00 |
|  | **#9** | 321631 | 690 | 441.55 | 1.56 | 1.57 | 60.14 | 0.00 |
|  | **#10** | 744000 | 1247 | 1021.40 | 1.22 | 1.23 | 23.74 | 0.00 |
|  | **#11** | 535507 | 925 | 735.17 | 1.26 | 1.26 | 22.97 | 0.00 |
|  | **#12** | 115851 | 242 | 159.05 | 1.52 | 1.52 | 18.68 | 0.00 |
| **Non-melanoma skin** | **#1** | 3073139 | 2045 | 1228.04 | 1.67 | 1.77 | 249.53 | 0.00 |
|  | **#2** | 875876 | 632 | 350.00 | 1.81 | 1.84 | 94.09 | 0.00 |
|  | **#3** | 27035 | 58 | 10.80 | 5.37 | 5.38 | 50.34 | 0.00 |
|  | **#4** | 4552453 | 2230 | 1819.18 | 1.23 | 1.26 | 49.40 | 0.00 |
|  | **#5** | 567716 | 373 | 226.86 | 1.64 | 1.66 | 40.02 | 0.00 |
|  | **#6** | 470391 | 296 | 187.97 | 1.57 | 1.59 | 26.75 | 0.00 |
|  | **#7** | 858485 | 484 | 343.05 | 1.41 | 1.42 | 26.29 | 0.00 |
|  | **#8** | 895004 | 484 | 357.65 | 1.35 | 1.36 | 20.59 | 0.00 |
|  | **#9** | 1841682 | 900 | 735.94 | 1.22 | 1.24 | 17.96 | 0.00 |
|  | **#10** | 535507 | 303 | 213.99 | 1.42 | 1.42 | 16.63 | 0.00 |
|  | **#11** | 418349 | 231 | 167.17 | 1.38 | 1.39 | 11.00 | 0.00 |
| **Thyroid** | **#1** | 7097258 | 4886 | 2629.12 | 1.86 | 2.29 | 1000.57 | 0.00 |
|  | **#2** | 875876 | 621 | 324.46 | 1.91 | 1.95 | 109.71 | 0.00 |
|  | **#3** | 964792 | 659 | 357.40 | 1.84 | 1.88 | 104.85 | 0.00 |
|  | **#4** | 278253 | 222 | 103.08 | 2.15 | 2.17 | 51.88 | 0.00 |
|  | **#5** | 27035 | 31 | 10.01 | 3.10 | 3.10 | 14.05 | 0.00 |
| **Stomach** | **#1** | 6206902 | 3373 | 2081.50 | 1.62 | 1.83 | 415.28 | 0.00 |
|  | **#2** | 2299120 | 1080 | 771.02 | 1.40 | 1.44 | 58.87 | 0.00 |
|  | **#3** | 224647 | 162 | 75.34 | 2.15 | 2.16 | 37.65 | 0.00 |
|  | **#4** | 813770 | 418 | 272.90 | 1.53 | 1.55 | 33.94 | 0.00 |
|  | **#5** | 88493 | 78 | 29.68 | 2.63 | 2.64 | 27.14 | 0.00 |
|  | **#6** | 80219 | 73 | 26.90 | 2.71 | 2.72 | 26.85 | 0.00 |
|  | **#7** | 45995 | 51 | 15.42 | 3.31 | 3.32 | 25.46 | 0.00 |
|  | **#8** | 270600 | 150 | 90.75 | 1.65 | 1.66 | 16.26 | 0.00 |
|  | **#9** | 2156671 | 851 | 723.25 | 1.18 | 1.19 | 11.33 | 0.00 |
| **Colon** | **#1** | 6903199 | 3333 | 2227.92 | 1.50 | 1.67 | 298.31 | 0.00 |
|  | **#2** | 875876 | 611 | 282.68 | 2.16 | 2.22 | 147.03 | 0.00 |
|  | **#3** | 1109803 | 572 | 358.18 | 1.60 | 1.63 | 55.81 | 0.00 |
|  | **#4** | 2680179 | 1123 | 864.99 | 1.30 | 1.33 | 37.98 | 0.00 |
|  | **#5** | 2096042 | 905 | 676.47 | 1.34 | 1.36 | 37.06 | 0.00 |
|  | **#6** | 27035 | 36 | 8.73 | 4.13 | 4.13 | 23.77 | 0.00 |
|  | **#7** | 80219 | 59 | 25.89 | 2.28 | 2.28 | 15.53 | 0.00 |
|  | **#8** | 115851 | 75 | 37.39 | 2.01 | 2.01 | 14.65 | 0.00 |
|  | **#9** | 321631 | 149 | 103.80 | 1.44 | 1.44 | 8.74 | 0.02 |
| **BNS** | **#1** | 8032309 | 1887 | 1559.75 | 1.21 | 1.28 | 41.17 | 0.00 |
|  | **#2** | 46321 | 47 | 8.99 | 5.23 | 5.25 | 39.80 | 0.00 |
|  | **#3** | 645733 | 215 | 125.39 | 1.71 | 1.74 | 26.85 | 0.00 |
|  | **#4** | 4552453 | 1080 | 884.01 | 1.22 | 1.26 | 23.16 | 0.00 |
|  | **#5** | 2096042 | 519 | 407.02 | 1.28 | 1.30 | 15.03 | 0.00 |
|  | **#6** | 224647 | 83 | 43.62 | 1.90 | 1.91 | 14.11 | 0.00 |
|  | **#7** | 121689 | 52 | 23.63 | 2.20 | 2.21 | 12.69 | 0.00 |
